# Clinical Psychology in Lithuania: Current Developments in Training and Legislation

**DOI:** 10.32872/cpe.v2i1.2835

**Published:** 2020-03-31

**Authors:** Evaldas Kazlauskas, Neringa Grigutyte

**Affiliations:** a Center for Psychotraumatology, Institute of Psychology, Vilnius University, Vilnius, Lithuania; University of Vienna, Vienna, Austria

**Keywords:** Lithuania, clinical psychology, legislation, training, education, policy

## Abstract

This paper presents an overview of the current status in training and legislation of clinical psychology in Lithuania. Clinical psychology training at the university level in Lithuania started soon after the collapse of the Soviet Union in the 1990s and was influenced by the social context and historical-political situation in the country. Currently, legislation for clinical psychology in Lithuania is in progress, and several promising regulations for psychology in health care were introduced in the last decade. However, psychologists, including clinical psychologists, are not licensed in Lithuania. The lack of legislation for psychology is the main obstacle for the recognition and establishment of clinical psychology in the country. In health care, the title ‘clinical psychologist’ is not common; ‘medical psychologist’ is the title used instead to refer to both clinical psychologists and health psychologists. We conclude that while the development of clinical psychology in Lithuania is promising, there is still a long way to go to establish clinical psychology as an important profession in Lithuania.

## Abstract (highlights)

- University training in clinical psychology started in the 1990s after the collapse of the Soviet Union.
- Legislation for clinical psychology in Lithuania is in progress and issues regarding the title and licensing of clinical psychologists are associated with the lack of regulation of psychology in the country in general.
- In Lithuania, the title ‘clinical psychologist’ is not used in health care, and the titles of psychologists or clinical psychologists are not protected by law.

Clinical psychology in the Baltic States remains unknown and somewhat of a ‘white zone’ on the global map of psychology. This paper aims to present the status of clinical psychology in one of the Baltic States – Lithuania, with a brief overview of the training and legislation for clinical psychology in the country. The current paper is an update of the previous reports on the history of Lithuanian psychology (Bagdonas, Pociute, Rimkute, & Valickas, 2008), and is an extension of the overview of Lithuanian clinical psychology published two decades ago (Gailiene, 2000) with a focus on current national developments in clinical psychology. Grounded on the development of clinical psychology in Lithuania this paper is informative in understanding the challenges and diverse pathways of establishing clinical psychology at a national level in different countries.

## Historical Background

Lithuania is a country with a population of around three million, situated in the North-Eastern part of Europe. It has been an EU member state since 2004, together with the other two Baltic States – Latvia and Estonia. Lithuania’s history is marked by occupations and fights for freedom. Established as the independent Republic of Lithuania after World War I in 1918, Lithuania was occupied by the Soviet army in 1940-1941, followed by Nazi occupation in 1941-1944, and Soviet occupation again in 1944-1990 (Eidintas, Bumblauskas, Kulakauskas, & Tamošaitis, 2015). Lithuania was one of the republics of the former Soviet Union until 1990. The political situation in the country during the Soviet Regime was very restrictive and oppressive. Political violence and oppression that lasted for decades during the Soviet regime resulted in a loss of a large proportion of the population (Eidintas et al., 2015). Narratives of historical traumas are still vivid in the majority of families living in the country (Kazlauskas, Gailiene, Vaskeliene, & Skeryte-Kazlauskiene, 2017; Kazlauskas & Zelviene, 2016). Furthermore, the memory of occupation and fights for freedom continue to have a profound impact on politics, socioeconomic situation, science, and culture in the country up to this day.

The development of clinical psychology in Lithuania was closely related to the political situation of 20^th^ century Europe. In Lithuania, as well as in other post-communist countries in the region, particularly in the former Soviet republics, psychology was restricted and oppressed by the Soviet regime (Gailiene, 2000). Despite negative attitudes held by the Soviet regime towards psychology, the growing interest in psychology resulted in the establishment of the Lithuanian Psychological Association (LPA) in 1958 (Bagdonas et al., 2008), with almost 300 founding members. The first professional five-year psychology diploma-training program in Lithuania was opened at Vilnius University in 1969, producing the first graduates of this psychology program in 1974. This program was focused on engineering and work psychology, as it was the only way it could be deemed acceptable by the Soviet regime (Gailiene, 2000).

Officially, when psychology training was launched in 1969, it was not possible to study or practice clinical psychology. However, since the very start of the psychology program at the University, psychology students were interested in clinical psychology and first psychologists managed to get positions and started to work in psychiatric hospitals in the 1970s (Bagdonas et al., 2008). During the 1970s and the 1980s, the field of clinical psychology was evolving through the initiatives of local professionals, as well as with the assistance of Lithuanian expats from the United States. During the Soviet era, U.S. psychology professors managed to sneak across the ‘Iron Curtain’ into Lithuania often under the pretense of visiting relatives and delivered clinical psychology training workshops and supervisions (Bieliauskas, 1977; Gailiene, 2000) which was a significant contribution to the development of clinical psychology at that time.

## Training in Clinical Psychology

### The Start of Clinical Psychology Training

A turning point in clinical psychology in Lithuania was a two-year master’s degree program in clinical psychology launched at Vilnius University in 1994, which marked the start of professional training of clinical psychologists’ in Lithuania. This ambitious aim to start the training of clinical psychologists was initiated by a group of psychologists from the Department of Psychology at Vilnius University who had previous interest in clinical psychology and psychotherapy and had relevant clinical experience. The master’s degree in clinical psychology program aimed to fulfill the needs of society to have professionally trained clinical psychologists.

Soviet legacy significantly impacted the training of clinical psychologists in Lithuania and the start of clinical psychology training was challenging. Clinical psychology research in Lithuania was almost non-existent during the Soviet occupation. Moreover, research methods and psychological assessment measures were not compatible with international standards due to the ‘Iron Curtain’ preventing the circulation of knowledge between the former Soviet Union and the rest of the World until the 1990s. Lithuania as part of the Soviet Union experienced even more restrictions in comparison to other Eastern and Central European post-communist countries outside of the Soviet Union (Gailiene, 2000). Access to international scientific knowledge of psychology, scientific papers, books, or modern assessment measures was restricted in the country until the 1990s. Thus, training in clinical psychology, especially in clinical psychological assessment, was significantly influenced by the Soviet approach to psychopathology and psychiatry. For example, psychological assessment training was focused on the use of Soviet cognitive assessment instruments, which were available at the time but not used outside of the Soviet Union.

### Current Clinical Psychology Training

Psychology training is currently regulated by the Ministry of Education and Science of the Republic of Lithuania which approved standards for training of psychology in 2015 (Ministry of Education and Science of the Republic of Lithuania, 2015). The national education standards in psychology are in line with the standards of the other EU member states and in accordance with the European Certificate in Psychology (EuroPsy) which was approved by the European Federation of Psychologists’ Associations (EFPA) (European Federation of Psychologists' Associations [EFPA], 2019; Lunt, Peiró, Poortinga, & Roe, 2014). Furthermore, the training of psychologists in Lithuania is based on Bologna regulations for higher education across Europe (Laireiter & Weise, 2019) and includes three cycles: bachelor’s degree, master’s degree, and doctoral degree.

Psychologists are trained at six universities in Lithuania. Bachelor’s degree programs in psychology take 3.5-4 years and master’s degree programs take two years to complete with a focus in various areas of psychology, such as clinical, health, educational, work and organizational, and forensic. Psychology degree programs offered at the universities are evaluated and accredited by the national agency responsible for the accreditation of all study programs in the country. LPA does not accredit psychology study programs; however, it was closely involved in the development of the national regulations for the training of psychologists.

The clinical psychology master’s degree program in Lithuania is a two-year program with 120 European Credit Transfer and Accumulation Study (ECTS) credits. Content of the program allows students to develop core competencies of clinical psychologists listed by the European Society for Clinical Psychology and Psychological Intervention (EACLIPT) Task Force on ‘Competences of Clinical Psychologists’ (EACLIPT Task Force On “Competences of Clinical Psychologists”, 2019). The majority of the study credits (67 ECTS credits) are dedicated to clinical psychology courses. Additionally, the master’s thesis research project is 30 ECTS credits, and supervised practice is 23 ECTS credits, which is a 4-month full-time internship in a clinical setting outside the University. The core courses of the curriculum are all clinical and include counselling skills training; adult and child clinical psychological assessment; introduction to the diversity of approaches in clinical psychology, with psychodynamic, existential, cognitive-behavioral and biopsychosocial approaches equally covered; developmental psychopathology; trauma and crisis psychology; and research methods in clinical psychology.

In Lithuania, around 30 students are admitted to the master’s program in clinical psychology annually, and the competition to enter the program is high. The admission numbers to master’s degree programs are regulated by the government, but the university and study program committees have the flexibility of establishing admission quotas based on the available resources each year. The majority of students in the clinical psychology program are funded by the state, with up to 30% of students being self-funded. By 2020, more than 400 clinical psychologists have graduated from the clinical psychology program in Lithuania.

For over 25 years, the master’s degree program in clinical psychology at Vilnius University remained the only training program for clinical psychologists in Lithuania. However, over the past few decades, other psychology master’s degree programs in the field of clinical and health psychology were launched in addition to the aforementioned clinical psychology program at a number of Lithuanian universities. Master’s study programs in health psychology were launched at Vilnius University, Vytautas Magnus University and the Lithuanian University of Health Sciences. Furthermore, the master’s degree program in counselling psychology was recently launched at Klaipeda University.

There are two four-year doctoral study programs in psychology in Lithuania, one at Vilnius University, and the other is a joint Ph.D. program of Mykolas Romeris University and Vytautas Magnus University. Up to 10 Ph.D. students are admitted annually to both of these programs. Around one-third of all Ph.D. students choose to conduct research in the clinical psychology field. However, as Ph.D. studies in Lithuania are research-based, Ph.D. students are expected to conduct research and publish papers, and no clinical training is included in the program.

## Legislation for Clinical Psychology

### Issues With the Use of the Title ‘Clinical Psychologist’

Due to negative attitudes by the Soviet regime towards clinical psychology and psychotherapy, psychologists were labeled ‘medical psychologists’ (Gailiene, 2000) since they started to work in health care institutions in the 1970s. All psychologists in national health care are still referred to as ‘medical psychologists’. Surprisingly, the term ‘medical psychologist’ persisted in Lithuania, and resulted in the title ‘clinical psychologist’ not existing. Thus, according to official statistics, there are zero clinical psychologists in Lithuania, but this is only because the term ‘clinical psychology’ is not used in the country’s health care system. There are, in fact, many graduates of clinical and health psychology programs who work in health care institutions or private practice across the country.

This attitudinal legacy from the Soviet era adds to the confusion in the legislation of clinical psychology in Lithuania. Despite the fact that the masters’ degree program in clinical psychology has existed for over 25 years, the profession of clinical psychology is not yet fully recognized or established in Lithuania. The titles ‘clinical psychologist’ and ‘psychologist’ in contrast to many other European countries are not protected. Furthermore, the title of clinical psychology is not used in psychology practice but only in training and education.

### Introduction of Regulation

There was no regulation for psychologists in health care in Lithuania until 2012 (Ministry of Health of the Republic of Lithuania, 2011). Moreover, there were no minimal training standards set in the field of clinical and health psychology prior to 2012. For decades, it was up to the employer to decide what training standards were considered as training standards to apply for psychologists in health care until the regulation was introduced. When it came to medical psychologists, health care institutions mostly used to employ psychologists with a five-year psychology diploma or master’s degree in any area of psychology, but occasionally psychologists with no more than a 4-year bachelor’s degree or even ‘professionals’ without a diploma could be employed before 2012.

It was only in 2012 that psychologists were included in the system of the Lithuanian national accreditation agency for health professions. Consequently, health care institutions could only hire registered medical psychologists with a master’s degree in health or clinical psychology. This new regulation was introduced with collaborative efforts between the Ministry of Health and LPA, which insisted that a bachelor’s degree in psychology and master’s degree with a specialization in health or clinical psychology should be a minimum requirement for psychologists to practice in health care. Several years of a transition period ensured that psychologists who started work before clinical and health psychology training became available in Lithuania and had substantial experience in clinical work could be registered as psychologists eligible to work in the health care setting.

This regulation did not include psychologists working outside the public health care setting, which is why psychologists providing psychological counselling or psychotherapy in private practice are not yet registered or regulated. Psychological services of registered medical psychologists in licensed health care institutions are reimbursed by the National health care insurance. However, due to the lack of staff and resources, access to psychologists’ services is restricted and there are long waiting lines. Psychologist’s services in private practice outside of health care institutions are not reimbursed by the National health care insurance. In reality, even non-professionals can declare themselves psychotherapists or clinical psychologists and start delivering services in private practice without any formal training in psychology in Lithuania. This is because law in Lithuania regulates neither the psychologist’s profession nor psychological services nor does it protect the psychologist’s title.

### Debates About the Regulation of Psychology

Legislation for clinical psychologists is part of the national regulation of psychology. Until 2020, psychology in Lithuania was not regulated by national laws and not included in the list of the licensed professions, except for school psychologists working in the national education system (European Parliament, 2016).

Debates about the standards for professional psychologists have been intense for over a decade. There are conflicting opinions among psychologists regarding the minimal training standards or regarding which institutions should license psychologists in Lithuania. Over the past decade, several proposals for a new law have been brought in the Lithuanian Parliament. These proposals included various requirements for minimal training, ranging from licensing psychologists for independent practice with only a bachelor’s degree in psychology to requirements of holding bachelor’s and specialized master’s degree in addition to having one-year experience of supervised practice in the field of intended practice, such as clinical and health psychology, educational psychology, or work and organizational psychology, which would be in line with the EFPA’s EuroPsy regulations (European Federation of Psychologists' Associations [EFPA], 2019). While most psychologists in Lithuania agreed that at least a master’s degree is needed to be granted a psychologist’s license, debates on the licensing agency still are ongoing. Proposals as to which organization should play the role of the licensing agency ranged from self-regulation of professionals by LPA to the establishment of a new Chamber of Psychologists or choosing one of the governmental institutions.

### Regulation of Psychologists in Health Care

Despite the lack of regulation on the national level, an important step for psychologists working in public health care was the document ‘Medical norm’ issued by the Ministry of Health of the Republic of Lithuania in 2018 (Ministry of Health of the Republic of Lithuania, 2018). This document defined the aim, the area of practice, and the methods of psychologists who work in the national health care system under the title of ‘medical psychologists’. However, the professional title ‘clinical psychologist’ was not included in this document. Up until 2020, there has been no clear distinction between health and clinical psychology in terms of regulation and fields of practice in health care. Current legislation in Lithuania allows for graduates of either health or clinical psychology master’s programs to work in health care in positions of psychologists in primary care, mental health care, prevention, rehabilitation, or in hospitals with patients who have somatic diseases. The regulation of medical psychologists in health care does not include differentiation between a child and adult psychologists, psychologists who provide assessment and those who mostly provide psychological counselling or use various methods of psychotherapy.

### Clinical Psychology, Psychotherapy, and Psychiatry

The present work focused solely on training and legislation for clinical psychology, therefore, it does not extend to the situation of psychotherapy in Lithuania. There are multiple psychotherapy schools that offer post-diploma training in various psychotherapy approaches in Lithuania, such as cognitive-behavioral, psychodynamic, child psychodynamic, group analysis, existential, gestalt, Jungian analysis, family therapy, and others. Training in specialized psychological therapies for posttraumatic stress disorder (PTSD), such as Eye Movement Desensitization and Reprocessing (EMDR), is also available in the country (Schäfer et al., 2018). Majority of psychologists who work in health care or private practice pursue psychotherapy training after having obtained a master’s degree from the university. However, there are no statistics available on how many psychologists have had additional psychotherapy training after the completion of psychology studies at university. The legal distinction between psychotherapy and clinical psychology remains unclear since law in Lithuania does not yet regulate psychotherapy. The relationship between psychiatry and clinical psychology is also not part of this paper. While these fields share a mutual interest in psychopathology and treatment of mental disorders, they also have a history of diverse interactions.

## Future Directions

This brief report presented struggles in establishing clinical psychology as a profession in Lithuania, a post-communist EU country. Our review demonstrated that the development of clinical psychology in Lithuania has been rather successful with a history of over 25 years of clinical psychology training available at the university level. Furthermore, regulations and standards for psychologists in health care have recently been introduced in Lithuania. However, our review also revealed controversies surrounding the use of the title ‘clinical psychologist’ and difficulties in establishing clinical psychology as an important field and profession in Lithuanian society.

Several future directions could be identified for further progress of clinical psychology in Lithuania:

- The term ‘clinical psychologist’ should be used officially to identify psychologists who provide services in health care and have training in clinical psychology.
- Continuing education in clinical psychology is needed to constantly update the knowledge of psychologists who work in Lithuania. Legislation and licensing of psychological practice should include a formal requirement for continuing education in the field of clinical psychology after university graduation.
- Training of clinical psychologists should be more focused on research. Potentially this could be achieved by more intense international collaboration and learning from countries that have more expertise in research and training in clinical psychology. The staff of clinical psychology programs could focus more on staff exchange with other international institutions to modernize training in Lithuania.
